# Ni–Mo nanostructure alloys as effective electrocatalysts for green hydrogen production in an acidic medium†

**DOI:** 10.1039/d4ra08619h

**Published:** 2025-01-15

**Authors:** Medhat M. Kamel, Alaa A. Abd-Ellah, A. Alhadhrami, Mohamed M. Ibrahim, Zeinab M. Anwer, Salah S. Shata, Nasser Y. Mostafa

**Affiliations:** a Department of Chemistry, Faculty of Science, Suez Canal University Ismailia 41522 Egypt n.mostafa@science.suez.edu.eg +201113343594; b Department of Chemistry, College of Science, Taif University P.O. Box 11099 Taif 21944 Saudi Arabia; c Geology Department, Faculty of Science, Suez Canal University Ismailia 41522 Egypt

## Abstract

Achieving a net-zero emissions economy requires significant decarbonization of the transportation sector, which depends on the development of highly efficient electrocatalysts. Electrolytic water splitting is a promising approach to this end, with Ni–Mo alloys emerging as strong candidates for hydrogen production catalysts. This study investigates the electrodeposition of Ni and Ni–Mo nanostructured alloys with high molybdenum content onto low-carbon steel cathodes using a novel alkaline green lactate bath. Catalyst morphology, microstructure, and composition were characterized using SEM, XRD, XPS, and EDX. Results showed molybdenum content increased with current density, ranging from 40.14 wt% at 1.12 mA cm^−2^ to 61.68 wt% at 5.56 mA cm^−2^, with average particle sizes of 39.4 nm for Ni, 20.7 nm for Ni–2Mo (56% Mo), and 30.8 nm for Ni–4Mo (65% Mo). The alloys comprised tetragonal MoNi_4_, metallic Ni, metallic Mo, and MoO_3_ phases. Ni–4Mo exhibited superior HER performance in 0.5 mol L^−1^ H_2_SO_4_, with the lowest Tafel slope (−113 mV dec^−1^), highest exchange current density (1.250 mA cm^−2^), and good stability after 250 cycles. It also outperformed Ni–2Mo at −50 mA cm^−2^, demonstrating its promise as a durable and efficient HER catalyst in acidic media.

## Abstract 2

Ni–4Mo demonstrating its promise as a durable and efficient HER catalyst in acidic media.

## Graphical abstract

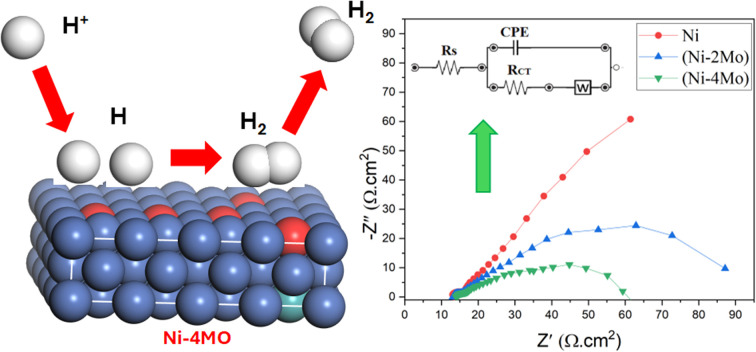

## 1. Introduction

One of the biggest issues facing our society today is addressing environmental sustainability and climate change while simultaneously fulfilling the ever-increasing need for energy.^1,2^ Because it has no emissions and possesses a high gravimetric energy density, hydrogen is considered one of the green fuels.^3^ High-quality hydrogen can be produced in an environmentally friendly and sustainable way using electrocatalytic water splitting.^2–4^ The hydrogen evolution reaction (HER) is a cathodic half-reaction of water splitting that reduces H^+^ ions or H_2_O molecules in acidic and alkaline solutions, respectively, to generate gaseous hydrogen.^5–8^

Platinum metal is the gold standard for HER electrocatalysts with a good yield of hydrogen. Nevertheless, the high cost and insufficient Pt reserves limit the wide range of commercial applications.^3,9^ As pure nickel (Ni) is inexpensive and readily available, it has been suggested as a Pt substitute.^10^ However, Ni shows inadequate electrocatalytic activity and gradual suppression toward HER because of its limited ability to desorb OH^−^ species and the formation of nickel hydride species.^11–13^ Many alloys based on Ni have been thoroughly investigated to improve HER performance. The use of metals and metals alloys supported on 2d nanostructures is a promising technique to enhance many electrocatalytic reactions.^14–16^ The ranking of the HER activity of Ni-based binary alloys is Ni–Mo > Ni–Zn > Ni–Co > Ni–W > Ni–Fe > Ni–Cr.^17^ The activity and the beneficial impacts of the two metals, Ni and Mo, have led to a great deal of work being put into the production and assessment of Ni–Mo alloys.^5^ The Ni–Mo catalyst showed potent resistance to corrosion, superior electrical conductivity, and good catalytic activity.^18,19^

Several techniques have been used to produce Ni–Mo alloys, including powder metallurgy, spraying, flame spraying, reduction, fusion, electrodeposition, and pulse electrodeposition.^20,21^ For mass production, electrodeposition is an easy, inexpensive, and low-temperature process.^22,23^ By adjusting the electrochemical deposition conditions, the electrodeposition approach allows for the control of the electrocatalysts' sizes, forms, and architectures on the surface of conducting materials. Another benefit of the electrochemical deposition process is that it doesn't require the use of a capping reagent, surfactant, or any other kind of dispersion agent.^24–26^

There has been a lot of interest in understanding the fundamentals of water electrolysis to produce green H_2_. Two half-reactions are typically involved in the process: the oxygen evolution reaction (OER) at the anode and HER at the cathode. Different types of catalysts can be used to speed up these two processes in acidic and alkaline conditions. Because of the higher ionic conductivity, faster H reaction dynamics, and lack of CO_2_ pollution, water electrolysis is much more beneficial in acidic media than in alkaline ones.^8,27,28^ This results in higher current densities and higher-purity H_2_. Electrolyzes working in acidic media often have current densities that are 4–5 times higher than those operating in alkaline media.^27^ Acidic electrolyzers are more stable against load-cycling and shutdowns, which is more significant.^29^ Consequently, a more promising method for producing H_2_ is water electrolysis in acidic media, despite the problems of electrolyzing corrosion in acidic media.

In a study conducted by Shetty *et al.*,^30^ nanocrystalline Ni–Mo alloy coatings were electroplated on a copper cathode within the current density range of 0.01–0.04 A cm^−2^. The catalytic activity of the alloys was examined in a 1.0 M KOH solution with respect to the HER and OER. The Ni–Mo alloys demonstrated high activity for HER and OER. Xu *et al.*^31^ achieved remarkable electrocatalytic efficiency by electrodeposited Ni–Mo alloy (28.2 wt% Mo) on a Cu metal at 0.12 A cm^−2^. Using a pulse plating technique, Han *et al.*^32^ produced Ni–Mo thin films with a highly amorphous structure on mild steel by utilizing 3.5 × 10^−2^ A cm^−2^ current density. The deposited alloys (21.6 wt% Mo) showed good electrocatalytic activity. A continuous 3D nanopatterned Ni–Mo solid solution catalyst was created by Kim *et al.*^33^ The synthesized catalyst acquired an overpotential of 39 mV at 10 mA cm^−2^ for HER in 1 M KOH. Cao *et al.*^34^ used electrodeposition technique in a gravity field to construct the 3D porous Ni–Mo film. The overpotential needed for obtaining 100 mA cm^−2^ for HER in 10% NaOH is less than 50 mV.

The electrodeposited Ni–Mo alloy film's surface was modified by the development of a surface sulphuration strategy.^35,36^ The characterization of the material has demonstrated the existence of NiS, which covers the Ni–Mo alloy's surface and causes a NiS/NiMo heterostructure to form. Electrochemical analyses indicated that the surface sulphuration resolved the HER degradation problem in addition to significantly increasing HER activity. Tang *et al.*^37^ produced different Ni-alloy HER catalysts using a one-step electrodeposition technique. With the help of the two-chamber electrolyzer system, the catalytic electrodes were isolated and optimized. After 70 hours of continuous operation, a voltage of 1.64 V at a current density of 200 mA cm^−2^ is achieved. Hydrothermally coupled H_2_ reduction was used by Wang *et al.*^38^ to create a low-overpotential NiMo/NMF electrode. Temperatures and periods of various hydrothermal and H_2_ reduction processes were investigated. With low overpotentials, the NiMo/NMF electrode displays impressive HER activity.

Most of the researchers used citrate baths to electrodeposit Ni–Mo alloys.^30,32,35,37^ The current investigation used a new alkaline lactate bath to electrodeposit Ni–Mo nanostructured alloy catalysts of varying compositions onto low-carbon steel cathodes. Lactate ions were chosen because of their high solubility in the plating bath. It is widely accessible, reasonably priced, and environmentally benign. Studies were conducted on the catalysts' morphology, microstructure, and chemical composition. Additionally, the durability, electrocatalytic stability, and HER performance of Ni–Mo catalysts were examined.

## 2. Experimental details

### 2.1. Preparation of catalysts

The Ni and Ni–Mo alloy coatings were electrodeposited onto steel coupons (3 cm × 3 cm × 0.05 cm) that were supplied by the Egyptian Iron and Steel Company in Egypt. Table 1 presents the chemical composition of steel coupons. The steel coupons were polished with silica suspension and ground to grit 1200. The next step was ultrasonic cleaning for 10 minutes in acetone and then ethanol. A solution contained 30 g L^−1^ of Na_2_CO_3,_ and 30 g L^−1^ of sodium triphosphate was then used to degrease the coupons. To eliminate any remaining oxide particles or scale, the samples were finally subjected to an acid pickling process at room temperature, immersing them in 75% HCl solution.^39^ An adhesive that was resistant to chemicals and nonconducting was used to cover the other side of the coupons. A 3 cm × 3 cm × 0.1 cm platinum plate, provided by Alfa Aesar in Germany, was used as the anode. The positive and negative poles of an electrical source were connected to the anode and cathode, respectively.

**Table 1:** Chemical composition of steel substrate, wt%

% C	% Si	% Mn	% P	% S	% Al	% Fe
0.08	0.01	0.03	0.025	0.025	0.045	The rest

An electrolytic bath containing hydrated nickel sulfate, ammonium molybdate, sodium sulfate, and lactic acid was used to electroplate the samples (Table 2). The Merck Company in Egypt supplied all the salts that were utilized. The bath's pH was 10, and the temperature was 333 K. The total metal content in the bath was kept constant at 0.05 mole L^−1^. In electrodeposition of Ni–Mo alloys, we used two baths with different ammonium molybdate concentrations (0.02 and 0.04 M). The impact of current density on the chemical composition the deposits with tested on bath with 0.04 M ammonium molybdate concentrations. Ni and Ni–Mo alloys were electrodeposited galvanostatically using a two-electrode cell. The cell was constructed from Perspex material in the form of a rectangle with a Pt anode and a steel cathode. For the deposition process, stagnant solutions were used. After a half-hour deposition period, the steel coupon was taken out, cleaned with distilled water, allowed to dry, and weighed. After the deposits were dissolved in 50% HNO_3_ acid and diluted with bi-distilled water, the alloy composition was determined using an atomic absorption spectrometer (Thermo, model: SOLAAR).

**Table 2:** Bath composition and working conditions of the electroplating process for Ni–Mo alloy

Composition and working conditions		Test samples		
		Ni	Ni–2Mo	Ni–4Mo
Bath composition	NiSO_4_·7H_2_O (M)	0.05	0.03	0.01
	(NH_4_)Mo_7_O_24_·4H_2_O (M)	—	0.02	0.04
	CH_3_CH(OH)COOH (M)	0.3	0.3	0.3
	Na_2_SO_4_ (M)	0.1	0.1	0.1
	Ammonia solution	Excess	Excess	Excess
Working conditions	pH	10	10	10
	Current density (mA cm^−2^)	2.77	2.77	2.77
	Deposition time (minutes)	30	30	30
	Temperature (K)	333	333	333

### 2.2. Characterization of catalysts

The microstructure and phase morphology were examined using a low vacuum SEM (JOEL JSM-6510 LV), and an EDX device attached to the SEM was used to determine the chemical analysis. To identify the type of phases present, the X-ray diffraction (XRD) technique was utilized with Cu radiation (*k*_α_ = 0.1540 nm). The XRD device (Rigaku miniFlex 600 diffractometer) was operated at 45 kV of voltage and 40 A of current.

The Scherrer's equation was used to calculate the crystalline size (*D*).^40^1
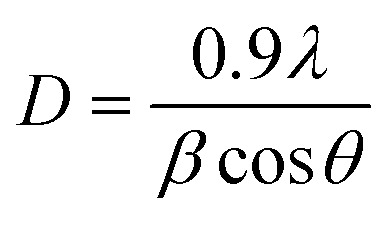
where *λ* is the X-ray wavelength (0.1540 nm), *β* is the full-width-of-half maximum (FWHM), and *θ* is the reflection angle.

### 2.3. Electrochemical tests

A typical three-electrode cell was utilized for the potentiodynamic polarization experiments. The coated samples worked as the working electrode, Ag/AgCl (3.0 M KCl) was the reference electrode, and Pt was the auxiliary electrode. The prepared Ni–Mo electrodes were coated with epoxy except for a circular working area of 3.0 mm diameter (area = 0.0707 cm^2^). Ni–Mo electrodes were exposed to 0.5 mol L^−1^ H_2_SO_4_ solution. By adjusting the working electrode potential between 0.0 and −1.5 V *vs.* Ag/AgCl, the polarization graphs were plotted. A scan rate of 50 mV s^−1^ was utilized. 5 mV sine waves were used to obtain EIS data over a frequency range of 1 MHz to 0.1 Hz. Before recording the impedance spectra, the coated steel electrode was left in the investigated solution at the OCP for 30 minutes. All electrochemical measurements were done with Palmsens4 potentiostat and EIS data was fitted by PSTrace software.

There are two approaches to assessing the electrocatalytic performance of Ni–Mo nanostructure electrodeposited alloys for HER. The first approach compares the potential of different Ni–Mo electrodeposited alloys as measured in linear polarization curves (*I*–*V*) at a constant current density of −50 mA cm^−2^. The second approach compares the slope of Tafel polarization curves' cathodic region (b-cathodic) and the exchange current density. Linear sweep voltammetry (LSV) testing with a scan rate of 50 mV s^−1^ was used to establish the electrocatalytic stability and durability of the fresh catalysts for 250 cycles. Chronopotentiometric analysis was then used to evaluate the catalyst's long-term stability for 24 hours at a constant current of −50 mA cm^−2^.

## 3. Results and discussion

### 3.1. The electrodeposition mechanism of the Ni–Mo alloy

In essence, understanding the mechanism gives researchers control over the deposition process and allows them to design new and improved alloys with specific functionalities. The impact of current density on the chemical composition of Ni–4Mo alloys was studied, and the results are given in Table 3. The table indicates that when the applied current density increases up to 2.77 mA cm^−2^, the Mo content increases as well. Refractory metal (Mo) content in the alloy increases when the parameters that speed up the mass movement of metal ions are increased. It follows that Mo deposition is governed by diffusion. This finding is closely linked to the electrodeposition of Ni–Mo from citrate baths, as they are the most frequently employed ligand in Ni–Mo electrodepositions.^41^ According to Table 3, this conclusion should also be applied in the present case (lactate baths), as in citrate and gluconate solutions,^42^ the Mo content changes in accordance with the current density. Thus, the mechanism of the Mo reduction in co-deposition with Ni in lactate baths follows the same trend as that with citrate ions suggested by Podlaha and Landolt,^41^ which was further validated by Zeng *et al.*^43^Eqn (2)–(7) provide and describe a conceivable mechanism for the electrodeposition of Ni–Mo alloys utilizing lactate ions (Lac) as a complexing agent.

2 Ni_(aq)_^2+^ + 2e^−^ → Ni_(s)_

3 Ni_(aq)_^2+^ + 2Lac_(aq)_^−^ → [Ni(Lac)_2_]_(aq)_

4 [Ni(Lac)_2_]_(aq)_ + MoO_4(aq)_^2−^ + 2H_2_O_(l)_ + 2e^−^ → [Ni(Lac)_2_(MoO_2_)]_ads_ + 4OH_(l)_^−^

5 [Ni(Lac)_2_(MoO_2_)]_ads_ + 2H_2_O_(l)_ + 4e^−^ → Mo_(s)_ + [Ni(Lac)_2_]_(aq)_ + 4OH_(l)_^−^

6 [Ni(Lac)_2_]_(aq)_ + 2e^−^ → Ni_(s)_ + 2Lac_(aq)_^−^

7 H_2_O_(l)_ + e^−^ → ½H_2(g)_ + OH_(aq)_^−^

**Table 3:** Effect of current density on the composition of the Ni–4Mo alloy

Expt.	Current density (mA cm^−2^)	Mo (wt%)
1	1.12	40.14
2	1.66	52.34
3	2.77	65.15
4	5.56	61.68

While Ni reduction is dictated by kinetics, Mo deposition is mostly governed by mass transfer.^44,45^ Consequently, an increase in current density led to an increase in the voltage between the cathode and anode electrodes, which in turn promoted the transport of [Ni(Lac)_2_] complexes to the cathode surface. However, because Ni–Mo alloys are highly electrocatalytic, H_2_ production on the cathode's surface promotes a rise in the Mo content of the alloy.^46^ Consequently, the reduction of metal ions onto the cathode and the subsequent production of alloys with greater Mo contents are encouraged by raising the current density because the hydrogen that is held on the cathode surface is more readily reduced to H_2_ and discharged into the atmosphere.

Nevertheless, the Mo content decreases, and Ni^2+^ reduction is preferred at current densities higher than 2.77 mA cm^−2^. This observation can be explained by the fact that Mo reduction is prevented by the coordination compounds that are produced between lactate and Ni^2+^ ions, which have low stability.^42^ The diffusion of Mo ionic species is inhibited when H_2_ production rises on the cathode surface because the energy utilized in the electrodeposition process is mainly employed for hydrogen evolution at a faster rate. This is because the applied current density is used for HER rather than for the reduction of Mo ions.^47^

### 3.2. Surface morphology, chemical composition and microstructure analyses

**Fig. 1:**
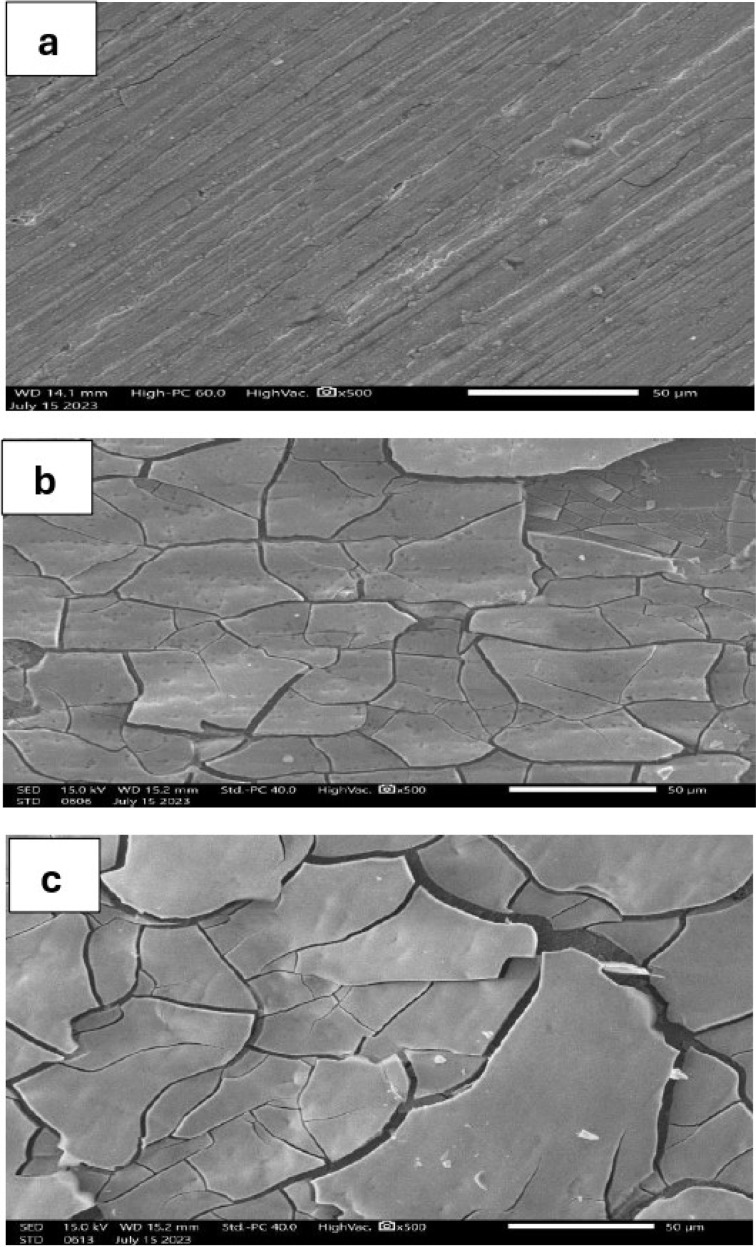
SEM micrographs of electrodeposited Ni (a), Ni–2Mo (b), and Ni–4Mo (c) alloys.

Fig. 1 shows the surface micromorphology of pure Ni, Ni–2Mo, and Ni–4Mo alloy coatings, prepared under the same deposition conditions as illustrated in Table 1. The pure Ni coating (Fig. 1a) completely covers the substrate surface with good adherence and appearance. The Ni–2Mo alloy coating contains 56% (wt.) Mo, and the Ni–4Mo alloy coating has 65% (wt.) Mo. The two alloy coatings exhibit cracks. Fig. 1b and c show that the steel surface is covered with a network of irregular microcracks and shows upheaval of micro-area edges. The same result was obtained by Zeng *et al.* for Ni–Mo–P alloys.^48^ Electrodeposited Ni–Mo alloys are prone to crack due to several factors related to the properties of molybdenum (Mo) and the electrodeposition process itself. The main causes are:

• Mo has a different atomic size and lattice structure compared to Ni. When a high concentration of Mo is incorporated into the Ni matrix during electrodeposition, it creates a mismatch in the atomic arrangement. This mismatch leads to internal stress within the deposited film, making it more susceptible to cracking.^49^

• During electrodeposition, H_2_ gas can evolve at the cathode, where the alloy is being deposited. Some of this hydrogen can become trapped within the Ni–Mo alloy. Hydrogen can weaken the interatomic bonds in the metal, further promoting crack formation.^50^

• At high Mo content, certain brittle intermetallic phases containing Mo may form within the deposit. These brittle phases can act as crack-initiation sites, leading to the propagation of cracks throughout the material.^51^

**Fig. 2:**
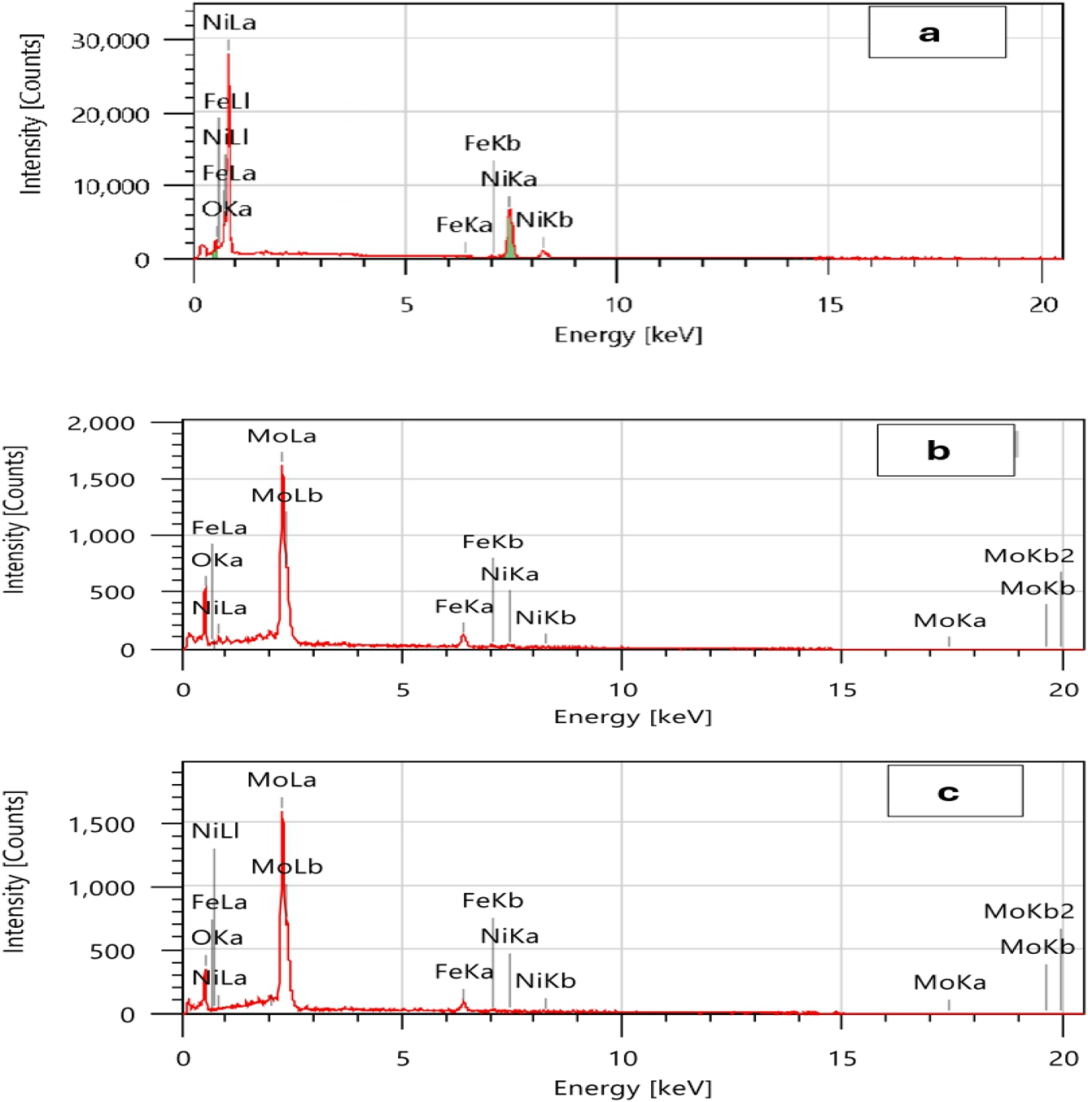
EDS spectra of electrodeposited Ni (a), Ni–2Mo (b), and Ni–4Mo (c) alloys.

Fig. 2 displays typical EDS spectra that describes the chemical composition of the coatings under investigation. The Ni coating (Fig. 2a) consists mainly of the three elements Ni (97.12 wt%), Fe (0.26 wt%), and O (2.62 wt%). The thinness of the deposited layer is shown by the existence of the Fe (substrate) peak. Oxygen is created by the oxidation of nickel. Winiarski *et al.*^52^ obtained the same results. In addition to Ni, Mo, and Fe, it was found that O is also present in a significant quantity on the coating surface of the deposited Ni–2Mo and Ni–4Mo alloy coatings.^53^ According to XRD, this is attributed to the formation of NiO and MoO_3_ compounds. The alloy coating Ni–2Mo (Fig. 2b) comprises a total of four constituent elements: Ni (3.11 wt%), Mo (56.21 wt%), Fe (10.81 wt%), and O (29.86 wt%). The Ni (0.94 wt%), Mo (65.15 wt%), Fe (9.23 wt%), and O (24.68 wt%) components are present in the Ni–4Mo alloy coating (Fig. 2c). Increased internal stress, surface pores, cracks, and other imperfections, as well as hydrogen evolution, are caused by a high Mo concentration.^54^

**Fig. 3:**
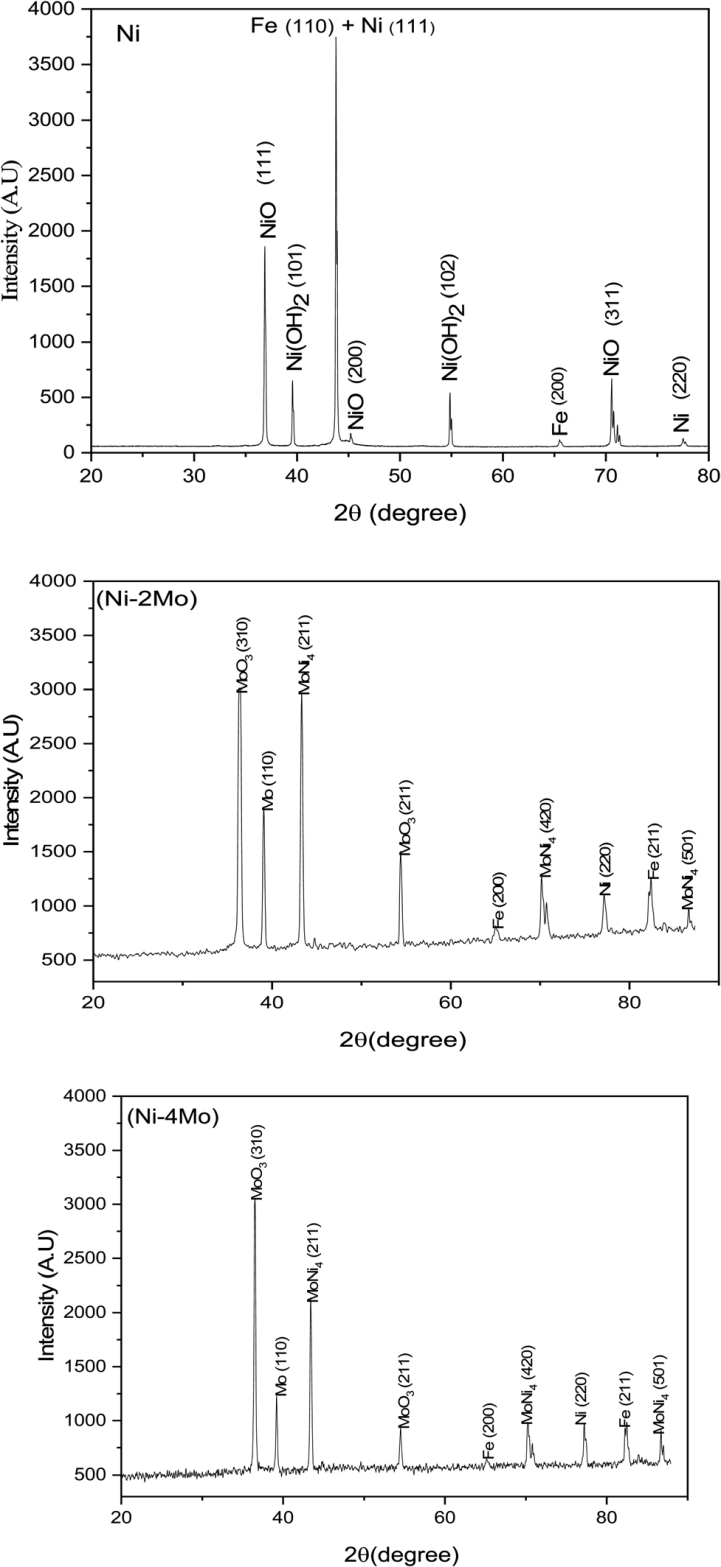
XRD patterns of electrodeposited Ni, Ni–2Mo, and Ni–4Mo alloys.

The XRD method was utilized to obtain the phase structure of the manufactured coatings. The XRD peaks of the Ni, Ni–2Mo, and Ni–4Mo coatings that were electrodeposited are shown in Fig. 3. The narrow peaks are a sign that the electrodeposits are crystalline. According to Sherrer's equation, the average particle sizes for the electrodeposited coatings (Ni, Ni–2Mo, and Ni–4Mo) are 39.4, 20.7, and 30.8 nm, respectively. The Ni coating's XRD pattern shows phases of Fe, β-Ni(OH)_2_, NiO, and metallic Ni. The metallic Ni phase can be identified by the planes of reflection (220) and (111) (JCPDS file 45-1027). The planes (311), (200), and (111) verify that the NiO phase has formed (JCPDS file 78-0643). According to JCPDS file 14-0117, the planes (102), and (101) are associated with the β-Ni(OH)_2_ phase formation. The JCPDS file 06-0696 shows that peaks (200) and (110) confirm the presence of the Fe substate. The Ni–2Mo alloy coating is composed of MoNi_4_, metallic Ni, metallic Mo, and MoO_3_, beside Fe of substrate. The tetragonal MoNi_4_ phase is represented by planes (501), (420), and (211) (JCPDS file 03-065-1533). The pure metallic Ni phase is designated by the plane (220). The MoO_3_ phase is confirmed by peaks (211) and (310) (JCPDS file 05-0508). The metallic Mo phase is represented by the plane of reflection (110) (JCPDS file 01-1208). The values of (200) and (211) confirm the presence of the Fe substate.

The XRD pattern of the Ni–4Mo alloy coating illustrates that the phases are identical to those found in the Ni–2Mo coating, suggesting that the phases are unaffected by the change in bath composition.

### 3.3. XPS of the electrocatalysts

The surface chemical compositions and valence states of the as-grown Ni, Ni–2Mo, and Ni–4Mo electrocatalysts explored by XPS as shown in Fig. S1–S3,† respectively. All electrocatalysts (Ni, Ni–2Mo and Ni–4Mo) show XPS spectrum for Ni 2p and O 1s. The Ni 2p_3/2_ region presents three peaks at about 851.1, 854.7, and 861.1 eV, respectively, which are associated with Ni^0^, Ni^2+^, and Ni^2+^ shakeup satellite peaks.^55^ The Ni 2p_1/2_ region presents two peaks at about 874.1 and 881.3 eV, respectively, which are associated with Ni^2+^, and Ni^2+^ shakeup satellite peaks.^56^ For the O 1s region, it contains two peaks at 529.2 eV and 531 eV attributed to oxygen vacancies in NiO, confirming the generation of NiO by the surface oxidation and also one peaks at 532 eV attributed to the surface hydroxyl groups.^55,57^

All Mo-containing electrocatalysts (Ni–2Mo and Ni–4Mo) as shown in Fig. S2 and S3,† show the XPS spectrum for Ni 2p, O 1s, and Mo (3d and 3p). The peaks at 231.1 eV and 235.2 eV are related to the Mo^6+^ (MoO_3_). Meanwhile, the peaks at 229.9 eV and 233.4 eV are attributed to the Mo^4+^ (MoO_2_). The Mo 3p core level spectra, shown in Fig. S2,† displays two main structures associated with the Mo 3p_3/2_ and Mo 3p_1/2_ levels which are split due to spin–orbit effects. In the Mo 3p_3/2_ region, the peak around 397.3 eV is assigned to Mo^4+^, but there is also a Mo^3+^ contribution near 394.5 eV. In the Mo 3p_1/2_ region, the peaks are near 415 and 413 eV, respectively, due to Mo^4+^ and Mo^3+^ ions.^58^

### 3.4. Electrocatalytic performance of deposited coatings towards HER

**Fig. 4:**
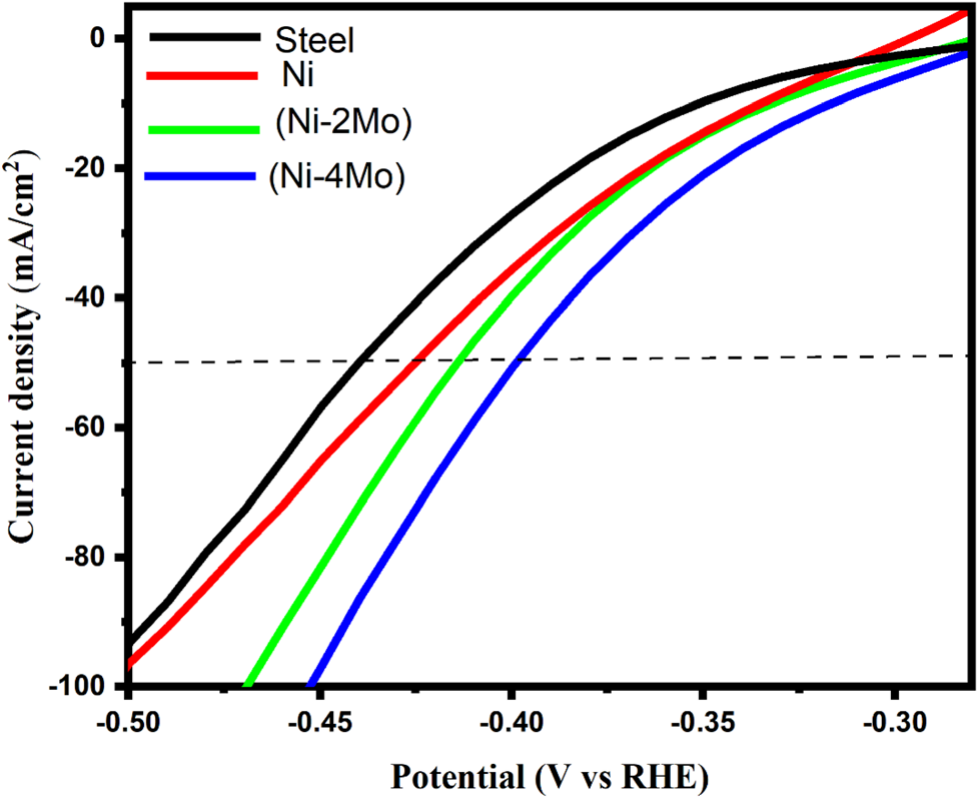
Polarization curves of the hydrogen evolution reaction on the deposited electrocatalytic materials in mol L^−1^ H_2_SO_4_ at 298 K. The potential scan rate is 50 mV s^−1^.

The polarization curves (*V*–*I*) of the hydrogen evolution reaction in an acidic solution (0.5 mol L^−1^ H_2_SO_4_) at 298 K were recorded to assess the electrocatalytic activity of the Ni–Mo alloy coatings under investigation (Fig. 4). In addition, Fig. 4 displays the hydrogen evolution polarization curves on a steel substrate and a pure nickel coating for comparison. It is feasible to assess the electrocatalytic capability of the investigated coatings through the locations of the curves in Fig. 4. The cathodic polarization of hydrogen evolution decreases with the increasing catalytic activity of the material. Undoubtedly, a decrease in cathodic polarization implies the shift of the related *I vs. E* curve towards greater positive values of the electrode potential. The results obtained are illustrated in Table 4. The onset potential of the steel substrate for HER is −390 mV (RHE); however, the onset potential of pure Ni coating is −360 mV (RHE). This result indicates that Ni metal coating has a higher catalytic activity for HER than steel substrate.

**Table 4:** The onset potential, the overvoltage at −50 mA cm^−2^, and the Tafel parameters of the hydrogen evolution reaction on the electrocatalytic materials under study

Sample	Onset potential (mV *vs.* RHE)	*η*_50_ (mV)	Tafel slope (*b*) (mVdec^−1^)	Exchange current density (mA cm^−2^)
Steel substrate	−390	−440	−142	0.054
Ni	−360	−420	−126	0.223
Ni–2Mo	−350	−410	−119	0.912
Ni–4Mo	−340	−390	−113	1.250

A considerable reduction in the overvoltage of the HER, or a rise in electrocatalytic activity, results from the addition of molybdenum to the nickel matrix (Fig. 4). There is a correlation between electrocatalytic activity and molybdenum content. Cathodic polarization decreases with increasing the Mo content of the electrodeposited material, *i.e.*, the electrocatalytic activity becomes more prominent. As we can see from Fig. 4 and Table 3, the Ni–4Mo alloy with a higher Mo content (65.15%) has a more pronounced catalytic activity for HER than the Ni–2Mo alloy with a lower Mo content (56.21%). The cause of that is Mo, a transition metal with partially filled d-orbitals. These orbitals play a crucial role in attracting and weakening the hydrogen bonds with the catalyst surface. This weakened bond allows for easier splitting of hydrogen molecules from water during the HER process.^59^ In addition, when Mo is incorporated into the nickel matrix, it might create specific sites at the atomic level where hydrogen evolution is more favorable. These sites could involve molybdenum atoms themselves or a combination of molybdenum and nickel atoms.^18^ At a current density of 50 mA cm^−2^, steel, nickel, Ni–2Mo, and Ni–4Mo requires an overpotential (*η*_50_) of −440, −420, −410, and −390 mV, respectively. Therefore, Ni–4Mo alloy with the highest Mo content exhibits the best electrocatalytic activity (Table 4).

**Fig. 5:**
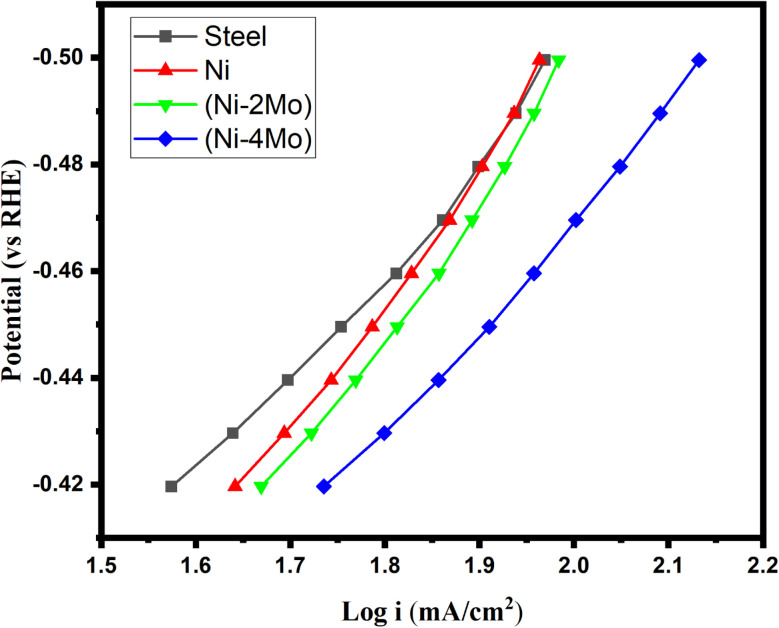
Tafel plots of hydrogen evolution reaction on the deposited electrocatalytic materials in mol L^−1^ M H_2_SO_4_ at 298 K.

Fig. 5, which was produced by fitting the linear polarization data of Fig. 4 to the Tafel equation, provided the electrochemical kinetic parameters utilized to characterize the HER reaction.

8 *η* = *b* log(*j*/*j*_o_)

where *η* and *j* represent the overpotential and the current density, respectively. *j*_o_ is the exchange current density, and *b* is the Tafel's slope. *j*_o_ measures of the rate of reaction at equilibrium, where the applied potential equals to the equilibrium potential. Conversely, a higher Tafel's slope indicates a slower reaction rate and a greater sensitivity of the overpotential to changes in current density. The Tafel slopes for steel, and Ni are −142 and −126 mV dec^−1^, respectively. This result is indeed an indicator of coupled reactions on the electrode surface, involving oxide, hydroxide, and/or hydride formation or oxide reduction.^60^ These processes are happening alongside hydrogen evolution, hindering the reaction, and requiring a larger overpotential to achieve the desired current density. On the other hand, the Tafel slope values close to −120 mV dec^−1^ are associated with Ni electrodes made from high-purity metal under carefully regulated circumstances, such as melting in an inert atmosphere without heat treatment.^61^

The exchange current density of the steel substrate is 0.054 mA cm^−2^, which falls within the typical range reported for steel in acidic media. It suggests some HER activity, but it is low compared to other materials. This means steel is not the most efficient catalyst for splitting hydrogen from water. *j*_o_ for Ni is 0.223 mA cm^−2^, which is slightly higher than steel, indicating improved HER activity for Ni. It is within the expected range for Ni in acidic media.

Based on the data in Table 4, among the materials listed, Ni–4Mo (with a higher Mo content) exhibits the best performance for the HER. The potential at which the current starts to significantly increase, indicating the onset of the electrochemical reaction. A lower onset potential indicates a smaller amount of energy required to initiate the HER. Ni–4Mo has the smallest negative onset potential (−340 mV *vs.* RHE), signifying its superior activity at lower applied potentials. Overpotential refers to the extra energy needed over the theoretical potential to motivate the reaction at a certain current density. Lower overpotential translates to better catalytic performance. Ni–4Mo demonstrates the most favorable value (−390 mV) at a current density of −50 mA cm^−2^. The Tafel slope (*b*) is the slope of the linear region of the log(current) *vs.* potential plot (Fig. 5), obtained from the LSV data. It provides information about the rate-determining step of the reaction. A smaller Tafel^'^s slope suggests a faster increase in current density with increasing overpotential, indicating a more efficient electrocatalyst. In this case, Ni–4Mo has the smallest Tafel slope (−113 mV dec^−1^), implying a more efficient HER process. *j*_o_ represents the theoretical current density at zero overpotential. The Tafel line (linear region of the log(current) *vs.* potential plot) is extrapolated to zero overpotential to estimate the exchange current density. A higher exchange current density signifies better intrinsic activity for the HER. Ni–4Mo exhibits the largest exchange current density (1.250 mA cm^−2^), supporting its superior electrocatalytic activity.

Therefore, combining these observations, Ni–4Mo emerges as the most promising electrocatalyst for the HER among the studied materials due to its lower onset potential, lower overpotential at a given current density, faster current rise with increasing overpotential, and higher intrinsic activity.

There is general agreement that a several-step electrochemical process occurs on the cathode's surface to cause HER.^61,62^ In particular, the following reactions cause the multi-step electrochemical process in acidic conditions:

9 M + H_3_O_(aq)_^+^ + e^−^ → M–H + H_2_O_(l)_ (Volmer)

10 M–H + H_3_O_(aq)_^+^ + e^−^ → H_2(g)_ + H_2_O_(l)_ + M (Heyrovsky)

11 2M–H → H_2(g)_ + 2M (Tafel)

The absorbed hydrogen atoms are denoted by the M–H, while the letter “M” stands for one of the electrocatalyst's vacant surface sites. The Volmer reaction [eqn (9)] in acidic solutions involves the reduction of H_3_O^+^ ion and the production of hydrogen intermediates (M–H). The chemical Tafel step [eqn (11)]^63^ and/or the electrochemical Heyrovsky step [eqn (10)] participate in the subsequent formation of H_2_. The ideal M–H bonds on the electrocatalysts should, in accordance with Sabatier's principle, be neither too strong nor too weak to promote the creation of M–H intermediates and the liberation of H_2_.^64^ According to the experimental findings (Table 4), we can conclude that the Heyrovsky step, which includes the regions with the Tafel slope of approximately −120 mV dec^−1^, is what limits the HER kinetics under conditions used in this work. Bao *et al.*^65^ achieved the same results.

**Fig. 6:**
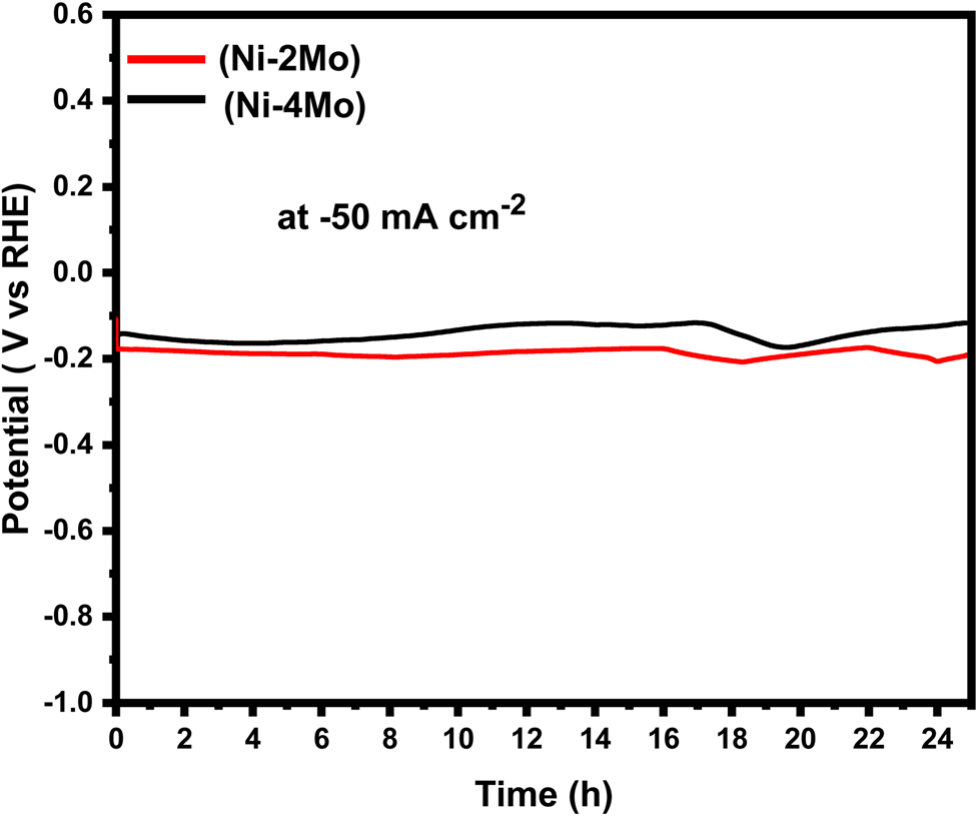
The chronopotentiometric curves for HER on the surface of Ni–2Mo and Ni–4Mo catalysts at a current density of −50 mA cm^−2^.

The electrocatalytic stabilities of the Ni–2Mo and Ni–4Mo coatings were evaluated by the chronopotentiometry (CP) technique. Chronopotentiometry is an electrochemical technique that measures the potential response of an electrode under constant current. For 24 hours, a steady current of −50 mA cm^−2^ was used for the CP investigation, and the potential of the working electrode was recorded as a function of time. The chronopotentiograms of Ni–2Mo and Ni–4Mo coatings are shown on Fig. 6.

Fig. 6 shows that, for both coatings, there is a slight initial fall in potential with time. This is because, at the start of the electrolysis process, the rapid application of current causes the reduction of hydrogen ions and the evolution of hydrogen gas to occur more quickly.^66^ 12 hours later, there was little to no change in the potential overtime, indicating that equilibrium was established. This suggests that H_2_ evolution occurs on the electrode surface successfully. It is worth observing that the potential of the Ni–4Mo curve is smaller than the potentials of the Ni–2Mo curve across the entire time range. This suggests that the Ni–4Mo coating is more efficient for HER at −50 mA cm^−2^ than the Ni–2Mo coating. A lower potential is required to drive the same current density through the Ni–4Mo coating, which is indicative of higher electrocatalytic activity.

**Fig. 7:**
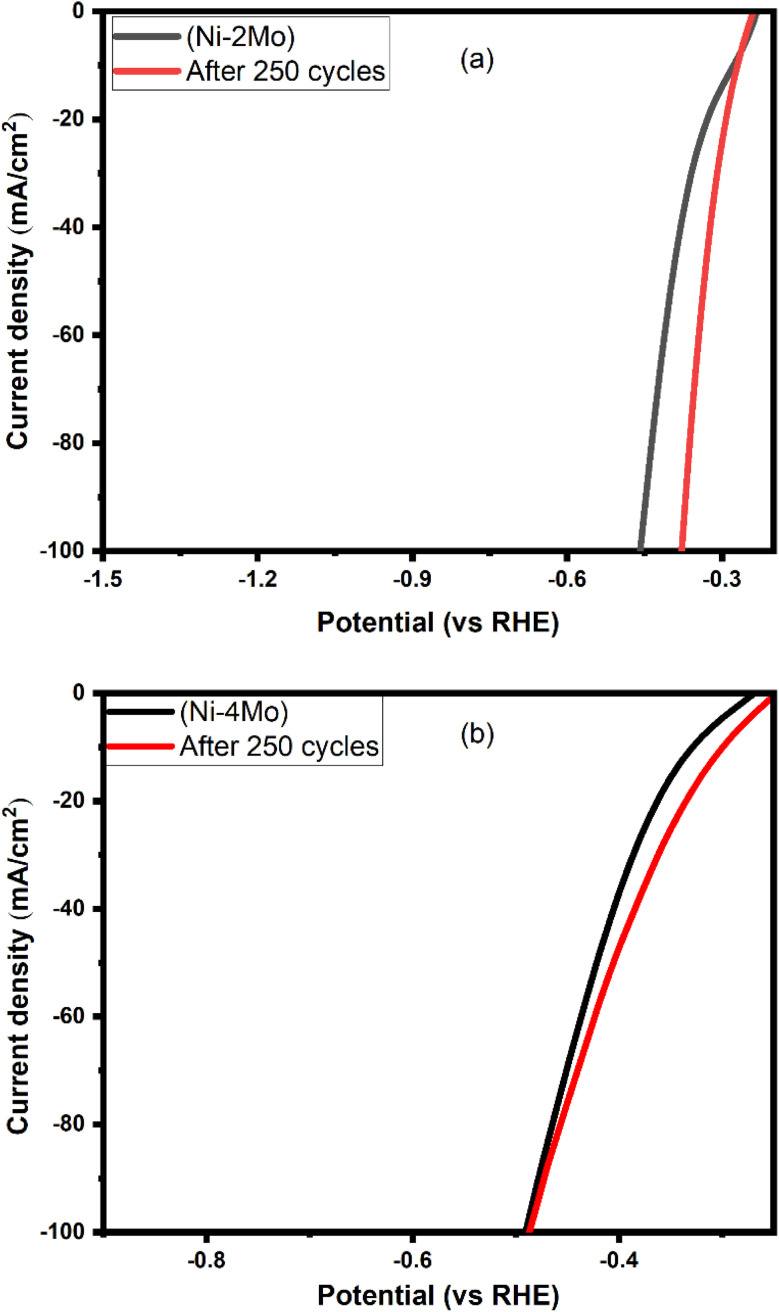
LSV for (a) Ni–2Mo fresh and after 250 cycles; (b) Ni–4Mo fresh and after 250 cycles. The curves are collected in 0.5 mol L^−1^ H_2_SO_4_ at 25 °C with a scan rate of 50 mV s^−1^.

LSV offers a straightforward and informative way to assess the stability of HER electrocatalysts. By analyzing the current density at relevant potentials for both fresh and aged catalysts, researchers can gain valuable insights into the catalyst's ability to perform consistently over time. To compare the stability of our top-performing Ni–Mo coatings over steel substrate, LSV collected at 0.5 mol L^−1^ H_2_SO_4_ at room temperature and a scan rate of 50 mV s^−1^ was used. Fig. 7a shows the LSV curves of the Ni–2Mo electrocatalyst for both the first cycle (fresh electrode) and after 250 cycles. The two curves appear to be roughly similar at potentials around −0.3 V *vs.* RHE, which is generally considered a benchmark potential for HER activity. This indicates that the Ni–Mo catalyst retains its ability to generate hydrogen at similar rates even after undergoing 250 cycles. It is worth observing a slight decrease in activity after 250 cycles. This is because the curve for the first cycle appears to have a slightly higher current density across a wider range of potentials compared to the curve after 250 cycles. Based on the LSV, the Ni–2Mo coating on the steel substrate appears to be a relatively stable catalyst for hydrogen evolution in 0.5 mol L^−1^ H_2_SO_4_.

For the (Ni–4Mo) electrocatalyst (Fig. 7b), while the current densities seem identical at −0.5 V *vs.* RHE, there is a slight decrease in activity after 250 cycles. This is because the curve for the fresh cycle appears to have a slightly higher current density across a wider range of potentials compared to the curve after 250 cycles.^67^ The rationale behind this is that, in contrast to the curve obtained after 250 cycles, the curve for the fresh cycle exhibits a little higher current density over a broader range of potentials. The LSV suggests that the Ni–4Mo coating demonstrates good stability for HER in 0.5 mol L^−1^ H_2_SO_4_, maintaining its catalytic activity to a significant degree after multiple cycles. After 250 cycles, XRD of the best electrocatalyst (Ni–4Mo) was characterized by XRD to investigate the phase change. Remarkably, all the metal oxides changed to metal alloys tetragonal MoNi_4_ phase (see Fig. S4†).

**Fig. 8:**
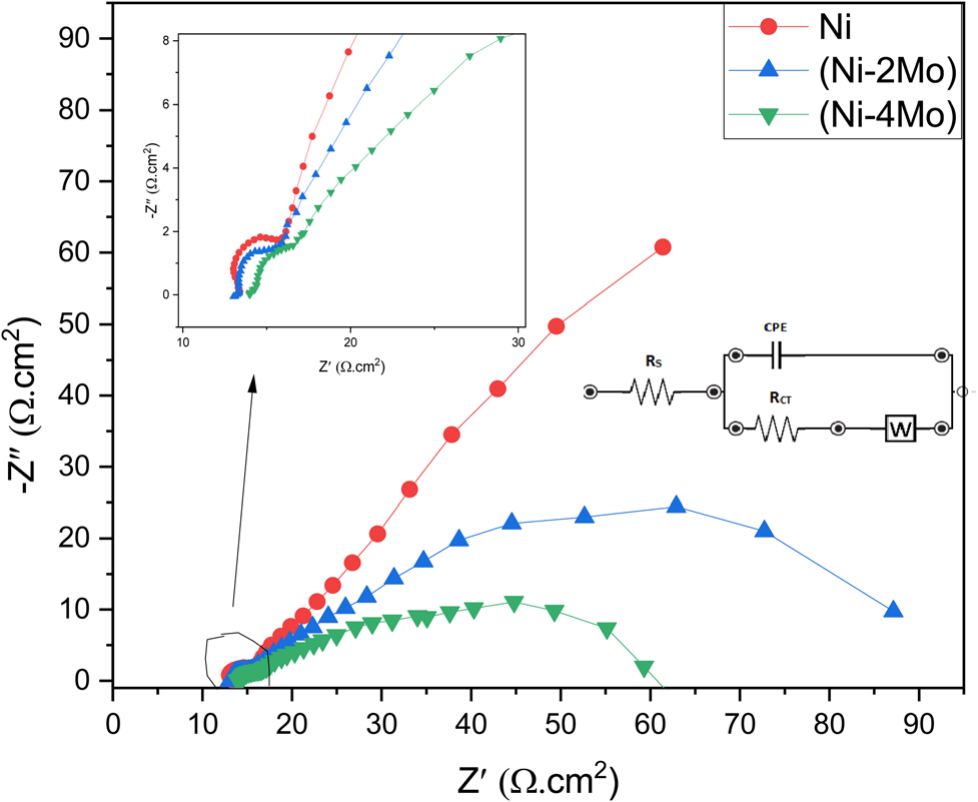
Measured (dots) and fitted (solid lines) Nyquist plots for Ni, (Ni–2Mo), and (Ni–4 Mo) coatings in 0.5 mol L^−1^ H_2_SO_4_. Insets: The equivalent circuit for EIS fitting; the locally enlarged plots for showing semicircles.

The EIS method was utilized to ensure a more comprehensive understanding of the processes involved in the HER of the studied Ni–Mo coatings.^68^Fig. 8 displays the electrodeposited (Ni–2Mo) and (Ni–4Mo) coatings' EIS responses under optimum conditions. An inset of Fig. 8 displays the simulated equivalent circuit. The simulated and measured impedance responses for coatings were found to be in close agreement. The coating equivalent circuit diagram is shown in the inset of Fig. 8 and Table 5 lists the values.

**Table 5:** Electrochemical parameters estimated from EIS data for Ni, Ni–2Mo, and (Ni–4Mo) coatings in 0.5 mol L^−1^ H_2_SO_4_

Sample	*R*_s_ (Ω cm^2^)	*R*_ct_ (Ω cm^2^)	*C*_dl_ (F cm^−2^)	*W* (Ω sec^−1/2^)
Ni	12.71	2.79	3.32 × 10^−6^	153.69
Ni–2Mo	13.23	2.08	5.98 × 10^−6^	130.18
Ni–4Mo	14.30	1.86	7.31 × 10^−6^	120.09

The semicircle at higher frequencies represents the charge transfer resistance (*R*_ct_) and the capacitance (*C*_dl_) of the electrode–electrolyte interface (Fig. 8). A smaller diameter indicates a smaller charge transfer resistance and faster electron transfer kinetics. The data in Fig. 8 suggests that the Ni–4Mo coating has the lowest charge transfer resistance, followed by Ni–2Mo and then Ni. This suggests that the Ni–4Mo coating is the most efficient catalyst for the HER in 0.5 mol L^−1^ H_2_SO_4_ among the three coatings tested. It is worth observing that the EIS data for HER on the Ni, Ni–2Mo, and Ni–4Mo coatings exhibit diffusion limitations (inset in Fig. 8). During HER, hydrogen ions (H^+^) need to diffuse through the electrolyte and reach the electrode surface. This diffusion process can introduce a frequency-dependent impedance, which the Warburg element helps represent in the equivalent circuit.

The equivalent electrical circuit depicted in the Fig. 8 inset was used to fit the EIS data. The solution resistance in the circuit is denoted by *R*_s_, the charge transfer resistance by *R*_ct_, CPE accounts for the capacitance at the electrode–electrolyte interface, and *W* represents the diffusion limitations within the electrolyte.

The interception of the Nyquist plot with the real axis at high frequencies corresponds to the value of *R*_s_. This is because at high frequencies, the capacitive elements (*C*_dl_) have negligible impedance, and the impedance is dominated by the resistive elements (*R*_s_ and *R*_ct_). The Nyquist plot often displays a semicircle, which is associated with the charge transfer process. The diameter of this semicircle is directly related to *R*_ct_.

The capacitance of the double layer (*C*_dl_) was computed by eqn (12)

12 *C*_dl_ = *Y*_o_(*ω*_max_)^*n*−1^

where *Y*_o_ is the coefficient of proportionality, *ω*_max_ = 2π*f*_max_, *f*_max_ is the frequency at which the imaginary component (*Z*_imag_) attains a maximum, and *n* is the shift in phase. The Warburg impedance, *W*, is often represented as:

13 *W* = *σω*^−1/2^

where *σ* is the Warburg coefficient, and *ω* is the angular frequency. The Warburg coefficient is related to the diffusion coefficient of the species involved in the electrochemical reaction.

According to the data in Table 5, one can conclude that:

• All three coatings have similar solution resistance values (12.71–14.31 Ω cm^2^), suggesting minimal differences in the overall resistance of the electrolyte enclosed between the working and reference electrodes.

• Ni–4Mo coating has the lowest *R*_ct_ (1.86 Ω cm^2^), implying potentially the highest intrinsic activity for HER among the three coatings.

• Ni–2Mo follows with a slightly higher *R*_ct_ (2.08 Ω cm^2^).

• Ni has the highest *R*_ct_ (2.79 Ω cm^2^), indicating potentially lower intrinsic activity compared to the Mo-containing coatings.

• Ni–4Mo has the highest *C*_dl_ (7.31 × 10^−6^ F cm^−2^), suggesting potentially a larger surface area or more accessible active sites compared to the other two coatings.^69^

• Ni–2Mo has a lower *C*_dl_ (5.98 × 10^−6^ F cm^−2^).

• Ni has the lowest *C*_dl_ (3.32 × 10^−6^ F cm^−2^).

• Ni–4Mo coating has the lowest *W* (120.09 Ω s^−1/2^), implying faster diffusion of the hydrogen ions within the electrolyte.

Overall, the EIS data suggests that the Ni–4Mo coating is a promising catalyst for the HER in 0.5 mol L^−1^ H_2_SO_4_.

**Fig. 9:**
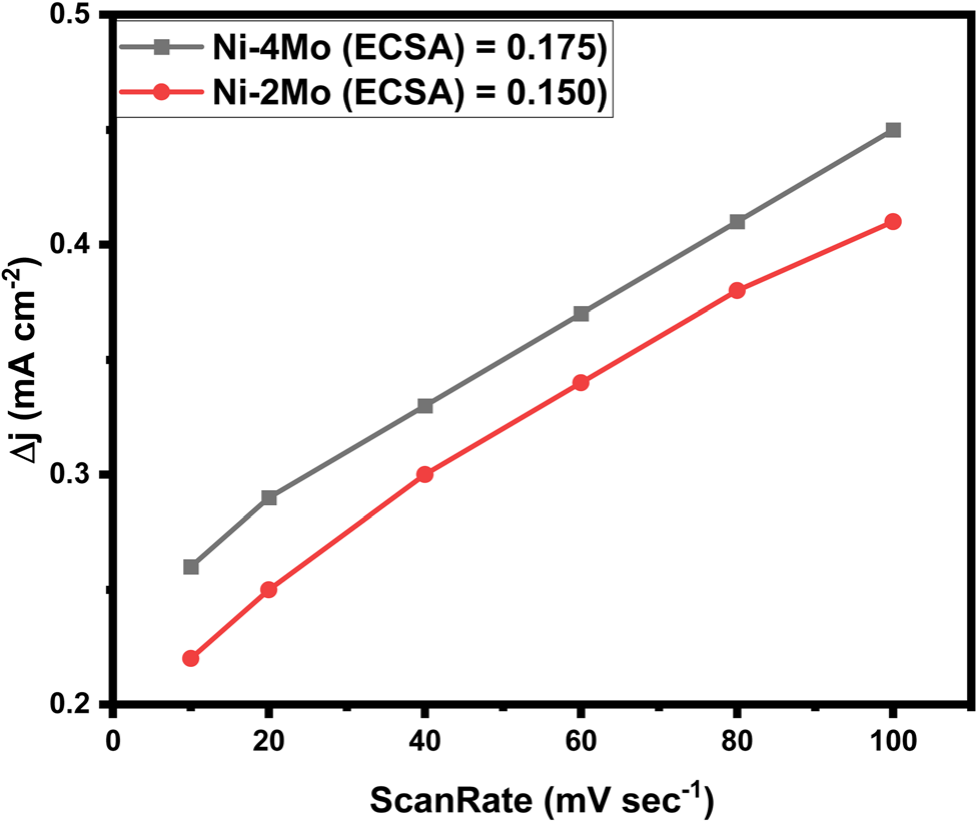
Plot of the capacitive current as a function of scan rates.

We measured the double layer capacitance (*C*_dl_) using CV curves in 0.5 mol L^−1^ H_2_SO_4_ at scanning rates ranging from 10 to 100 mV s^−1^ to support the opinions. This allows for the plotting of cathodic current against CV scanning rates. The slopes of the linear plots that were fitted match the *C*_dl_ values. The following eqn (14) ^70^ can be used to estimate the electrochemical active surface area (ECSA):14
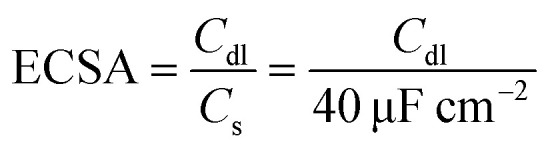
where *C*_s_ is the specific capacitance, which for a flat electrode ranged from 20 to 60 μF cm^−2^.^71,72^ A *C*_s_ value of 40 μF cm^−2^ was selected for this investigation.^73^Fig. 9 shows the *C*_dl_ values of the examined catalysts. It can be observed that the *C*_dl_ value of the Ni–4Mo catalyst (0.175) is greater than that of the Ni–2Mo catalyst (0.150), suggesting that the former contains a significantly greater number of activity sites. A higher ECSA value generally indicates a more efficient catalyst. This is because a larger surface area allows for more contact between the catalyst and the reactants, leading to faster reaction rates.

Table 6 compares the catalytic performance of our electrocatalysts (Ni–2Mo and Ni–4Mo) with other electrocatalysts that were reported in the literature. It is obvious from the reported data that the electrocatalysts investigated have good electrochemical activity towards hydrogen evolution from acidic medium.

**Table 6:** Comparison between the performance of the investigated electrocatalyst with the other electrocatalysts reported in the literature

Electrocatalyst	Overpotential (*η*) (V)	Tafel slope (−*b*) (mV dec^−1^)	Exchange current density (*i*_o_) (A cm^−2^)	References
Ni, 99.5%, forged	—	214	5.5 × 10^−6^	74
Ni–Mo(NF-D)/Ni, 22 at% Mo, DC plat.	*η*_200_ = −0.78	203	8.7 × 10^−6^	75
Ni–Mo(Han-P)/Ni, 23 at% Mo, P. plat.	*η*_200_ = −0.70	196	4.1 × 10^−6^	75
Ni–Mo, melted in inert atm., 25 at% Mo	—	152	1.1 × 10^−5^	76
Ni–Mo, arc melt. in Ar atm., 25 at% Mo	—	148	3.2 × 10^−5^	77
Ni–Mo/mild steel, 22 at% Mo, P. plat.	—	196	1.2 × 10^−2^	78
Co(OH)_2_@P–NiCo-LDH 226 134 42	*η*_10_ = −0.226	134	—	79
Ni/NiO@C	*η*_10_ = −0.204	137	—	80
Ni–2Mo/steel	*η*_50_ = −0.410	119	9.12 × 10^−4^	This work
Ni–4Mo/steel	*η*_50_ = −0.390	113	1.25 × 10^−3^	This work

## 4. Conclusions

Ni–Mo nanocrystalline catalysts were electrodeposited on low-carbon steel cathodes from an environmentally friendly lactate bath. The deposition was carried out in solutions containing nickel sulfate, ammonium molybdate, lactic acid, sodium sulfate, and ammonium hydroxide. The study examined the Ni–Mo alloy catalysts' microstructure, morphology, chemical composition, and electrocatalytic performance towards HER in an acidic medium (0.5 mol L^−1^ H_2_SO_4_). A high Mo content caused fissures to appear in the deposited alloys. The Mo content increased from 40.14 to 61.68 wt% as the applied current density increased from 1.12 to 5.56 mA cm^−2^. The electrodeposited alloy coatings (Ni–2Mo, and Ni–4Mo) had average particle sizes of 20.7 and 30.8 nm, respectively. The formed nanocomposite catalysts composed of MoO_3_, tetragonal MoNi_4_, metallic Ni, and metallic Mo. Superior electrocatalytic activity was implied by the alloy Ni–4Mo, which had the lowest Tafel slope (−113 mV dec^−1^) and the maximum exchange current density (1.250 mA cm^−2^). The presence of metal oxides of Mo and Ni may contribute to the electrocatalytic activity of the catalysis.^6,81^ Based on the data from linear sweep voltammetry (LSV), it was shown that the Ni–4Mo coating exhibited strong stability for HER in acidic solutions, retaining a notable level of catalytic activity even after 250 cycles. Chronopotentiometry findings showed that the Ni–4Mo coating is more effective than the Ni–2Mo coating for HER at −50 mA cm^−2^. The EIS measurements suggested that the Ni–4Mo coating was a potentially effective catalyst for the HER in 0.5 mol L^−1^ H_2_SO_4_. According to the experimental findings, the Heyrovsky step limits the HER kinetics under the conditions used in this work.

## Data availability

The data supporting the findings of this study are available within the article and its ESI.†

## Conflicts of interest

There are no conflicts to declare.

## Supplementary Material

RA-015-D4RA08619H-s001
