# Correction: Ni–Mo nanostructure alloys as effective electrocatalysts for green hydrogen production in an acidic medium

**DOI:** 10.1039/d5ra90064f

**Published:** 2025-05-27

**Authors:** Medhat M. Kamel, Alaa A. Abd-Ellah, A. Alhadhrami, Mohamed M. Ibrahim, Zeinab M. Anwer, Salah S. Shata, Nasser Y. Mostafa

**Affiliations:** a Department of Chemistry, Faculty of Science, Suez Canal University Ismailia 41522 Egypt n.mostafa@science.suez.edu.eg +201113343594; b Department of Chemistry, College of Science, Taif University P.O. Box 11099 Taif 21944 Saudi Arabia; c Geology Department, Faculty of Science, Suez Canal University Ismailia 41522 Egypt

## Abstract

Correction for ‘Ni–Mo nanostructure alloys as effective electrocatalysts for green hydrogen production in an acidic medium’ by Medhat M. Kamel *et al.*, *RSC Adv.*, 2025, **15**, 1344–1357, https://doi.org/10.1039/D4RA08619H.

The authors would like to clarify the affiliation list of the published article to correctly show where the work was conducted. In the original article, the affiliations at the time of publication of one author (Salah S. Shata) were listed as Department of Chemistry, College of Science, Taif University, P.O. Box 11099, Taif 21944, Saudi Arabia and Geology Department, Faculty of Science, Suez Canal University, Ismailia, 41522, Egypt. However, the only correct affiliation for this author is Geology Department, Faculty of Science, Suez Canal University, Ismailia, 41522, Egypt. The corrected list of affiliations at the time of publication of the original article is presented here.

The Royal Society of Chemistry apologises for these errors and any consequent inconvenience to authors and readers.

