# Supplementary material for: Ni–Mo nanostructure alloys as effective electrocatalysts for green hydrogen production in an acidic medium
Source: RSC Adv. 2025 Jan 15;15(2):1344–57. doi: 10.1039/d4ra08619h (PMC11734743; doi:10.1039/d4ra08619h)
Supplement: RA-015-D4RA08619H-s001 [file RA-015-D4RA08619H-s001.pdf]

Supplementary Information (SI) for RSC Advances.
This journal is © The Royal Society of Chemistry 2025

Supplementary information

Ni-Mo nanostructure alloys as effective electrocatalyst for green hydrogen production in an acidic medium

Medhat M. Kamel [1*], Alaa A. Abd- Ellah[1], A. Alhadhrami[2], Mohamed M. Ibrahim[2], Zeinab M. Anwer[1] , Salah S. Shata[2], Nasser Y. Mostafa[1]

[1]Department of Chemistry, Faculty of Science, Suez Canal University, Ismailia, 41522, Egypt.
[2]Department of Chemistry, College of Science, Taif University, P.O. Box 11099, Taif 21944, Saudi Arabia
[3]Geology Department, Faculty of Science, Suez Canal University, Ismailia, 41522, Egypt

XPS of the electrocatalysts

XPS of Ni coating

To elucidate the surface electronic states of Ni and O species in Ni sample, XPS characterization was performed. As shown in Fig. S1, the Ni $2p_{3/2}$ region presents three peaks at about 851.1, 854.7, and 861.1 eV, respectively, which are associated with Ni^0 , Ni^{2+}, and Ni^{2+} shakeup satellite peaks [1]. The Ni $2p_{1/2}$ region presents two peaks at about 874.1 and 881.3 eV, respectively, which are associated with Ni^{2+}, and Ni^{2+} shakeup satellite peaks [2]. For the O 1s region, it contains two peaks at 529.2 eV and 531 eV attributed to oxygen vacancies in NiO, confirming the generation of NiO by the surface oxidation and also one peaks at 532 eV attributed to the surface hydroxyl groups [1, 3].

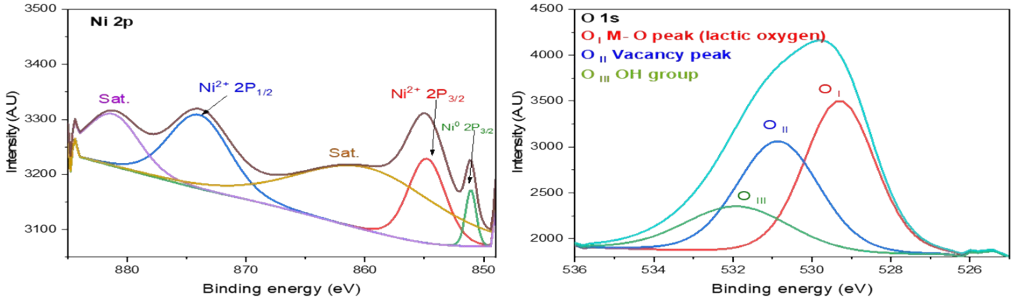

Fig. S1: XPS of Ni coating

XPS of Ni-2Mo coating

The surface chemical compositions and valence states of the as-grown Ni-2Mo catalyst explored by XPS. Fig. S2 show the XPS spectrum for Ni 2p, O 1s, and Mo (3d and 3p), respectively. The main peak at 853.9 eV as presented in Fig. S2 is attributed to metallic Ni. Additionally, the peaks at 856.5 eV, 873 eV and 876 eV demonstrate the existence of Ni^{2+} (NiO). For the O 1s region, it contains two peaks at 529.7 eV and 530.5 eV attributed to oxygen vacancies and one peak at 531.5 eV attributed to the surface hydroxyl groups. On the other hand, it can be found in Fig. S2 that the peaks at 231.1 eV and 235.2 eV are related to the Mo^{6+} (MoO_3). Meanwhile, the peaks at 229.9 eV and 233.4 eV are attributed to the Mo^{4+} (MoO_2) [1-5]. The Mo 3p core level spectra, shown in Fig S2, displays two main structures associated with the Mo $3p_{3/2}$ and Mo $3p_{1/2}$ levels which are split due to spin-orbit effects. In the Mo $3p_{3/2}$ region, the peak around 397.3 eV is assigned to Mo^{4+}, but there is also a Mo^{3+} contribution near 394.5 eV. In the Mo $3p_{1/2}$ region, the peaks are near 415 and 413 eV, respectively, due to Mo^{4+} and Mo^{3+} ions [6].

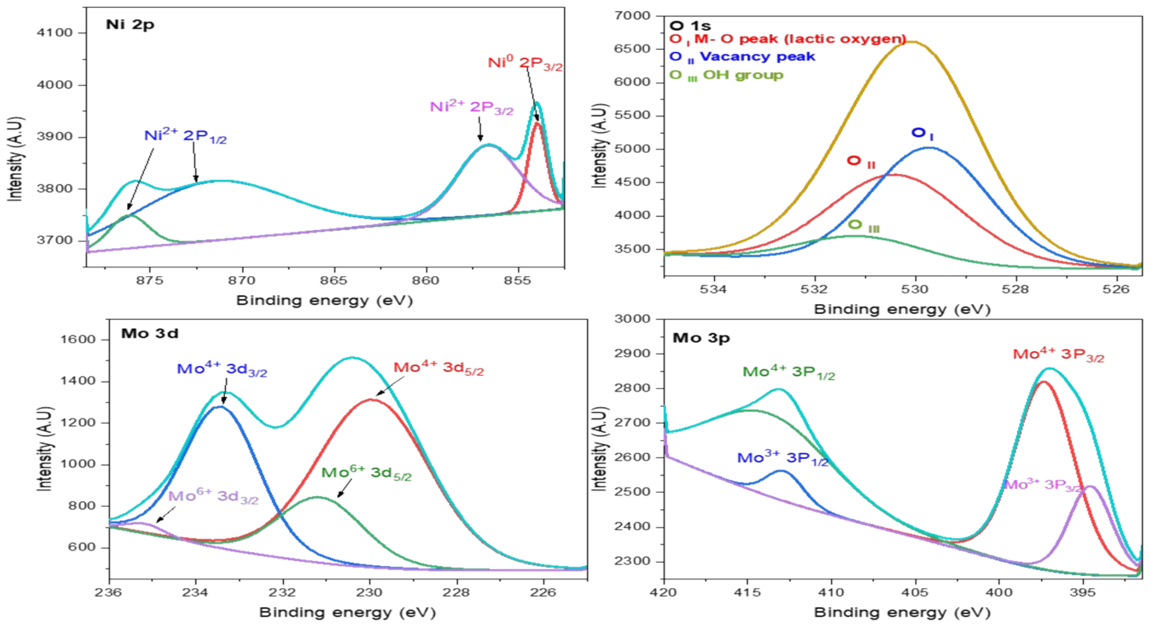

Fig. S2: XPS of Ni-2Mo coating

XPS of Ni-4Mo coating

The surface chemical compositions and valence states of the as-grown Ni-4Mo catalyst explored by XPS. Fig. S3 shows the XPS spectrum for Ni 2p, O 1s, and Mo (3d and 3p),

respectively. The main peak at 854.5 eV and one satellite peak at 860.1 eV as presented in Fig. S3 are attributed to Ni^{2+}. Additionally, the main peak at 873.7 eV demonstrate the existence of Ni^{2+} (NiO) and also peak at 870 eV are related to metallic Ni^0. For the O 1s region, it contains two peaks at 529 eV and 530.5 eV attributed to oxygen vacancies and also one peak at 532 eV attributed to the surface hydroxyl groups. On the other hand, it can be found in Fig. S3 that the peaks at 229.3 eV are related to the metallic Mo. Meanwhile, the two peaks at 230.3 eV and 233.6 eV are attributed to the Mo^{4+} (MoO_2). Moreover, the one peak corresponding to Mo^{6+} (MoO_3) is also found at 232.2 eV [1-5]. The Mo 3p core level spectra, shown in Fig S3, displays two main structures associated with the Mo $3p_{3/2}$ and Mo $3p_{1/2}$ levels which are split due to spin-orbit effects. In the Mo $3p_{3/2}$ region, the peak around 396.9 eV is assigned to Mo^{4+}, but there is also a Mo^{3+} contribution near 395.6 eV. In the Mo $3p_{1/2}$ region, the peaks are near 415 and 413 eV, respectively, due to Mo^{4+} and Mo^{3+} ions [6].

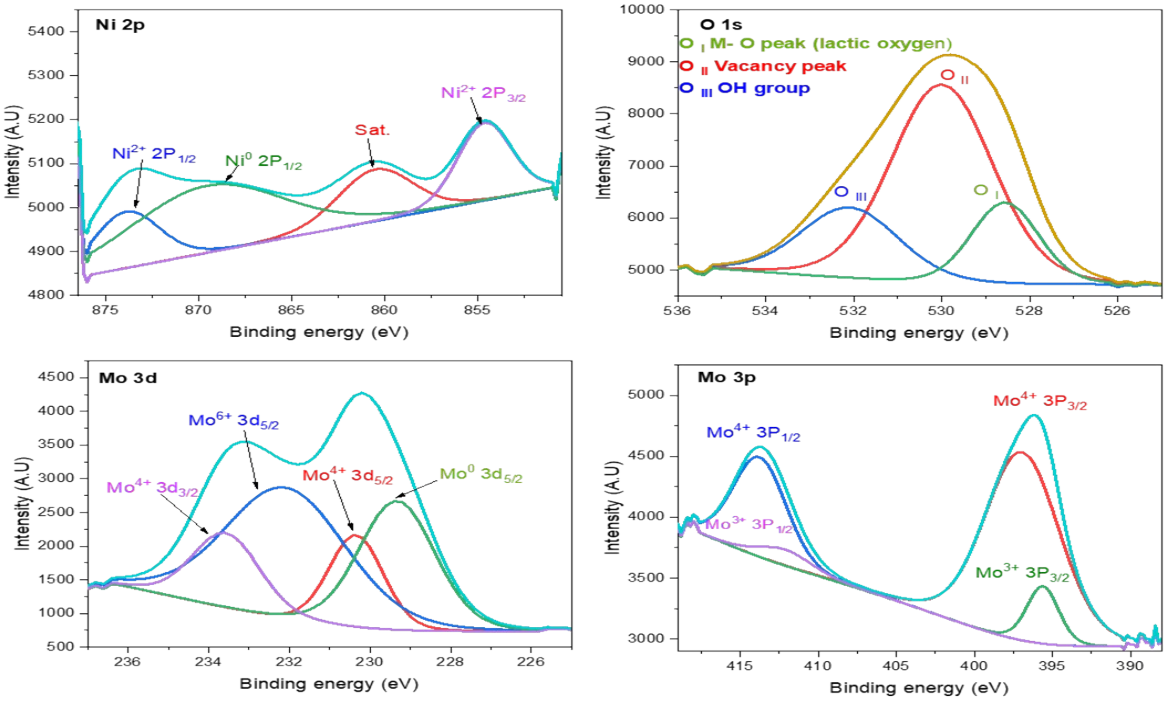

Fig. S3: XPS of Ni-4Mo coating

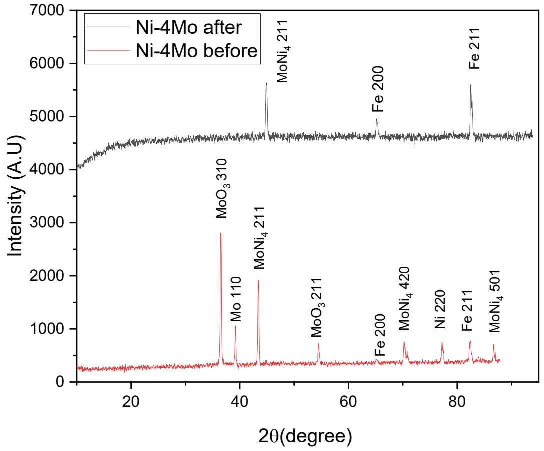

Fig. S4: XRD of Ni-4Mo catalyst before and after stability test.

Reference

1. Y. Zhang, G. Fan, L. Yang, L. Zheng and F. Li, *ACS Sustainable Chemistry & Engineering*, 2021, **9**, 11604-11615.
2. M. Y. Gao, C. Yang, Q. B. Zhang, J. R. Zeng, X. T. Li, Y. X. Hua, C. Y. Xu and P. Dong, *Journal of Materials Chemistry A*, 2017, **5**, 5797-5805.
3. X. Shi, X. Zheng, H. Wang, H. Zhang, M. Qin, B. Lin, M. Qi, S. Mao, H. Ning, R. Yang, L. Xi and Y. Wang, *Advanced Functional Materials*, 2023, **33**.
4. J. Sun, B. Yu, F. Tan, W. Yang, G. Cheng and Z. Zhang, *International Journal of Hydrogen Energy*, 2022, **47**, 15764-15774.
5. S. Sajjad, C. Wang, X. Wang, T. Ali, T. Qian and C. Yan, *Nanotechnology*, 2020, **31**.
6. L. M. S. Alves, S. S. Benaion, C. M. Romanelli, C. A. M. dos Santos, M. S. da Luz, B. S. de Lima, F. S. Oliveira, A. J. S. Machado, E. B. Guedes, M. Abbate and R. J. O. Mossanek, *Brazilian Journal of Physics*, 2015, **45**, 234-237.
